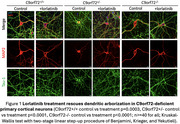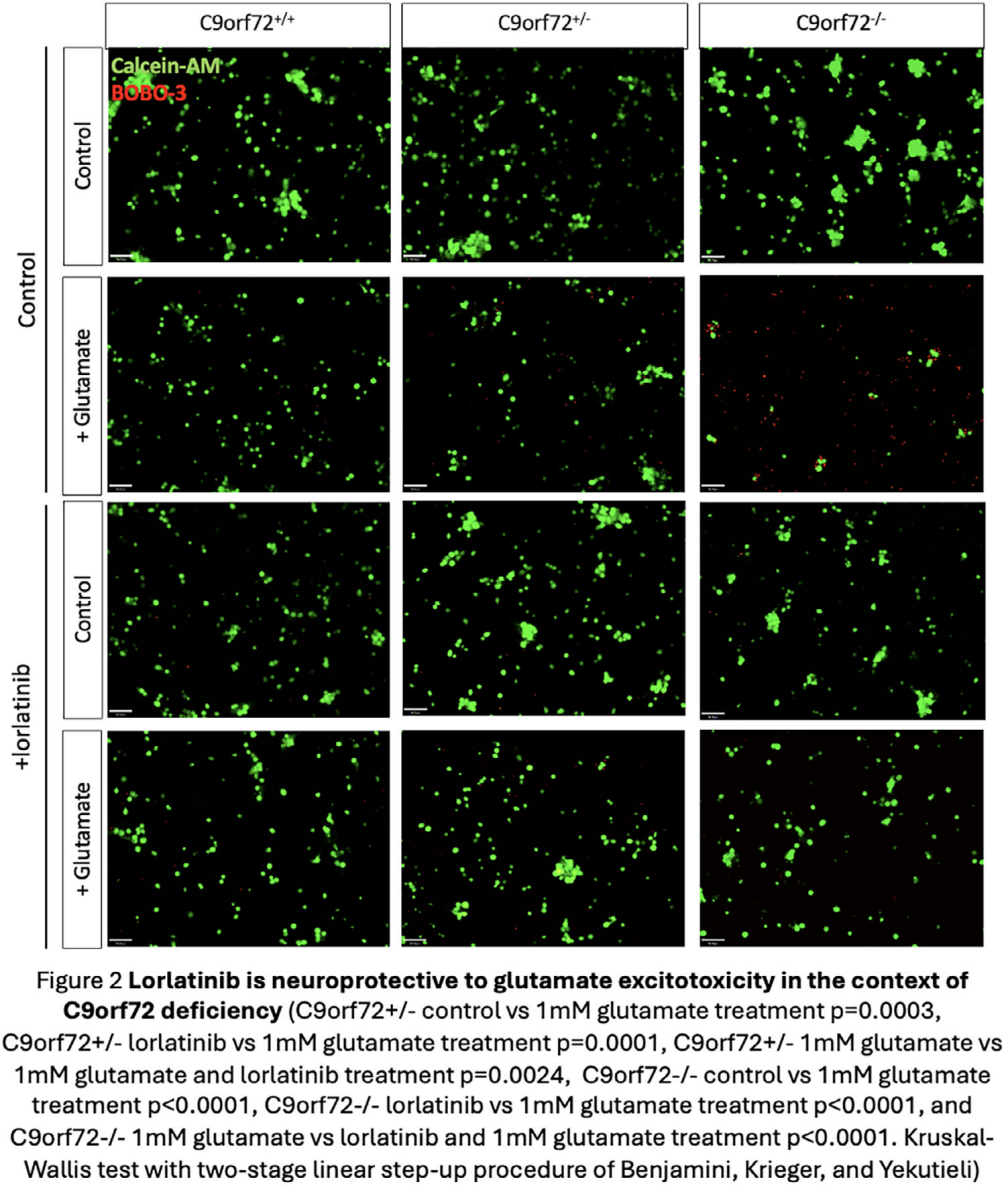# Targeting anaplastic lymphoma kinase (ALK) alleviates synaptic dysfunction associated with C9orf72 haploinsufficiency

**DOI:** 10.1002/alz70855_099957

**Published:** 2025-12-23

**Authors:** Bryan Kartono, Liliana Attisano, Janice Robertson

**Affiliations:** ^1^ University of Toronto, Toronto, ON, Canada; ^2^ Tanz Centre for Research in Neurodegenerative Diseases, Toronto, ON, Canada

## Abstract

**Background:**

Hexanucleotide repeat expansions in C9orf72 are the most common genetic cause of frontotemporal dementia (FTD) and amyotrophic lateral sclerosis (ALS). The repeat expansions cause C9orf72 haploinsufficiency and reduced C9orf72 protein expression. C9orf72 haploinsufficiency disrupts normal synaptic function, causing alterations in neuronal morphology, glutamatergic imbalances, and dysregulation of pre‐ and post‐synaptic proteins, linked to aberrant actin dynamics. These disruptions are central to the pathogenesis of C9orf72‐related FTD.

Lorlatinib, an ALK inhibitor used in cancer therapy, has known effects on regulating actin dynamics, acting through the PI3K‐LIMK‐cofilin pathway. Here, we explored lorlatinib as a potential therapeutic by testing its effects on rescuing neuronal phenotypes caused by C9orf72 haploinsufficiency.

**Methods:**

To assess lorlatinib's potential in mitigating synaptic dysfunction associated with C9orf72 haploinsufficiency, we used primary cortical neurons from C9orf72+/‐ mouse embryos (E13–16). We focused on dendritic arborization and vulnerability to glutamate excitotoxicity. Analyses were performed at DIV12–15 using immunocytochemistry and immunoblots to evaluate dendritic complexity and protein expression.

**Results:**

Primary cortical neurons with C9orf72 haploinsufficiency exhibit alterations in dendritic branching, indicating disrupted neuronal connectivity. Additionally, C9orf72 haploinsufficiency increases neuronal vulnerability to excitotoxicity and cell death under stress, evidenced by reduced viability following high glutamate exposure. Glutamate excitotoxicity is a common pathomechanism in neurodegenerative diseases, including FTD/ALS. This can be explained by elevated calcium‐permeable AMPAR subunit GluA1 levels at post‐synaptic sites, increasing vulnerability. These synaptic dysfunction phenotypes arise from aberrant activity of interrelated PI3K/Akt and LIMK1/cofilin pathways. Importantly, inhibition of ALK by lorlatinib rescues dendritic complexity and enhances neuronal resilience to glutamate‐induced damage by normalizing PI3K/Akt and LIMK1/cofilin activity. Lorlatinib may mitigate synaptic dysfunction and preserve neuronal connectivity, addressing key pathomechanisms underlying C9orf72‐FTD.

**Conclusion:**

Our findings highlight the therapeutic potential of lorlatinib, an ALK inhibitor, in mitigating synaptic deficits caused by C9orf72 haploinsufficiency. We emphasize the role of synaptic pathology in C9orf72‐FTD pathogenesis and suggest that targeting ALK with drugs like lorlatinib could offer promising therapeutic strategies for treating synaptic dysfunction caused by C9orf72 haploinsufficiency.